# New indication area in intersphincteric resection: transanal total mesorectal excision combined with intersphincteric resection

**DOI:** 10.1093/gastro/goad007

**Published:** 2023-03-08

**Authors:** Bülent Cavit Yüksel

**Affiliations:** Department of Colon & Rectum, General Surgery, Ankara Bilkent City Hospital, University of Health Science, Ankara, Turkey

##  

We read the article by Liu *et al*. [1] entitled “Transanal total mesorectal excision combined with intersphincteric resection has similar long-term oncological outcomes to laparoscopic abdominoperineal resection in low rectal cancer: a propensity score-matched cohort study.” The article is very interesting and boldly depicted. However, I noticed some areas of the article that I could not find the answers to.

The intersphincteric resection (ISR) method applied as sphincter-sparing was standardized by Rullier *et al*. [2]. Rullier *et al*. [3] did not prefer the ISR method in case of tumor invasion in the intersphincteric area. However, in this article, the authors applied the ISR method to intersphincteric space invasion. What is exciting and pleasing here, however, is that the oncologic outcomes and local recurrence rates are the same as in patients undergoing abdominoperineal resection.

Questions I want to ask in this article are as follows.

Question 1. How many patients had tumor invasion in the intersphincteric space?

Question 2. Is there a relationship between patients who developed local recurrence (LR) and tumor invasion in the intersphincteric area? Could this relationship be checked with univariate and multivariate analysis?

Question 3. What kind of surgeries were performed in patients who developed LR?

Question 4. This project was designed as a propensity score-matched cohort study. Although the demographic and clinical characteristics of the two groups of patients showed a homogeneous distribution, the fact that lymphovascular invasion, perineal invasion, and extramural vascular invasion, which have an impact on poor prognosis, were not examined creates a question mark.

Obviously, it is important to get adequate and satisfactory answers to these questions. It will lead us to prefer the ISR method in case of tumor invasion in the intersphincteric area. This is an achievement in terms of sphincter-sparing surgery.

### Conflict of Interest

None declared.

Authors’ reply

Zhi-Hang Liu and Liang Kang

Department of Colorectal Surgery, The Sixth Affiliated Hospital, Sun Yat-sen University, Guangzhou, Guangdong, P. R. China

We are glad that many doctors share our concern about the treatment of low rectal cancer. Under the premise of ensuring the survival of patients, we have been seeking for many years to preserve the anus of patients with low rectal cancer, in order to improve the quality of life of patients.

In this study, through preoperative data matching, some patients who could only receive abdominoperineal resection (APR) surgery received transanal total mesorectal excision (taTME) + intersphincteric resection (ISR) surgery under the new concept. The seemingly bold approach reflected our surgical team's confidence in the surgical concept and technology. The following are our answers for the questions.

Question 1. How many patients had tumor invasion in the intersphincteric space?

Answer: After matching, 100 patients in each group were enrolled in the subsequent study. We found that 66 patients in the laparoscopic abdominoperineal resection (laAPR) group had tumor invasion in the intersphincteric space compared with 64 patients in the taTME + ISR group.

Question 2. Is there a relationship between patients who developed LR and tumor invasion in the intersphincteric area? Could this relationship be checked with univariate and multivariate analysis?

Answer: During the follow-up period, seven patients in each group developed LR. Meanwhile, in both groups of patients with LR, four patients were found to have tumor-invaded intersphincteric space. Therefore, we do not find a relationship between patients who developed LR and tumor invasion in the intersphincteric space.

Question 3. What kind of surgeries were performed in patients who developed LR?

Answer: If patients had only LR, local resection was recommended by doctors. When patients had LR and distant metastasis, many patients were only willing to accept ostomy surgery and chemotherapy, or even only chemotherapy, rather than surgical treatment.

Question 4. This project was designed as a propensity score-matched cohort study. Although the demographic and clinical characteristics of the two groups of patients showed a homogeneous distribution, the fact that lymphovascular invasion, perineal invasion, and extramural vascular invasion, which have an impact on poor prognosis, were not examined creates a question mark.

Answer: In order to balance the differences between groups, preoperative data were usually used for matching, whether in our retrospective or prospective studies. At the same time, under similar preoperative conditions, we could compare the quality of surgical excision between the two groups.

For factors such as lymphovascular invasion, perineal invasion, and extramural vascular invasion, the previous imaging methods were not clear enough. In most cases, pathological state could only be known from the surgical specimen. Therefore, when conducting matching studies, we still consider gender, age, tumor size, preoperative TNM stage, and so on.

Our team will continue to publish research on the treatment of rectal cancer. Please pay attention to our subsequent studies. I hope that we can keep in touch with readers and make progress together.
